# Comparative risk-benefit profiles of different femoral drilling techniques in anterior cruciate ligament reconstruction: A protocol for a systematic review and network meta-analysis

**DOI:** 10.1097/MD.0000000000020544

**Published:** 2020-06-05

**Authors:** Ning Wang, Zhenglei Zhu, Ziying Wu, Hongyi He, Haochen Wang, Wei Li, Dongxing Xie, Yilun Wang

**Affiliations:** Department of Orthopaedics, Xiangya Hospital, Central South University, Changsha, Hunan, China.

**Keywords:** anterior cruciate ligament, anteromedial portal, outside-in, transtibial

## Abstract

**Background:**

Anterior cruciate ligament (ACL) injury experiences about 200,000 isolated cases annually, and ACL reconstruction has become the gold standard for the restoration of stability and functionality. In view of that incorrect graft placement is a common cause of ACL reconstruction failure, it is critically important to ensure that the tibial and femoral tunnels are properly placed during the operation. Therefore, we intend to conduct a network meta-analysis to comparatively evaluate the clinical outcomes among the different surgical techniques in ACL reconstruction.

**Methods:**

Embase, PubMed, and Cochrane Library will be searched through to retrieve the relevant literature up to April 2020. The outcomes include the International Knee Documentation Committee subjective/objective score, Lachman test, Lysholm score, laxity of knee joint, pivot-shift test, Tegner activity scale, and the number of adverse events. A Bayesian hierarchical framework will be used to evaluate the comparative efficacy among different fixation devices. Cochrane Q test and I^2^ statistics will be applied to evaluate the heterogeneity, and the Cochrane risk of bias assessment tool will be employed to evaluate the study quality and the risk of bias.

**Results:**

The comparative risk-benefit profiles of different femoral drilling techniques will be evaluated based on the existing evidence, in order to summarize a prioritization regimen.

**Conclusion:**

Findings from this network meta-analysis will provide useful reference to patients, surgeons, and guideline makers in the related fields.

**Registration:**

Open Science Framework (OSF) Preregistration. April 20, 2020. osf.io/uzahs

## 1 Introduction

Anterior cruciate ligament (ACL) injury is the most commonly-seen ligament injury of the knee joint, which experiences about 200,000 isolated cases annually in America.^[1]^ In most situations, surgery is recommended for the treatment of ACL injury. More specifically, ACL reconstruction is currently the gold standard for the restoration of stability and functionality, with high rates of return to preoperative activities and low rates of reoccurrence.^[2,3]^ Statistically, there are more than 100,000 patients receiving ACL reconstruction every year in the United States.^[4,5]^ The focus of ACL reconstruction has been shifting toward the proper placement of tibial and femoral tunnels at present, because incorrect graft placement has been recognized as one of the major causes of ACL reconstruction failure.^[6,7]^

Arthroscopic ACL reconstruction techniques can be classified into 3 main types: transtibial (TT), anteromedial portal (AMP), and outside-in (OI) techniques.^[8]^ In the past 20 years, the TT technique has long been considered the standard method for drilling a femoral tunnel through the tibial tunnel in ACL reconstruction, and has generally achieved satisfactory clinical outcomes.^[9]^ However, the femoral tunnel drilled through the tibia bone tunnel may not provide anatomically appropriate results.^[10]^ Some patients still presented a lack of rotational control due to a more vertical graft orientation from a higher femoral tunnel aperture position after receiving surgery with the TT technique.^[11]^

Owing to the benefit of increased obliquity of the coronal graft, the AMP and OI techniques, which are collectively known as the independent drilling (ID) technique, have been drawing a growing interest.^[12,13]^ As the location of the femoral tunnel is independent of the tibial tunnel, both techniques can produce an anatomically positioned femoral tunnel more easily and provide better rotational stability and anterior translation than the TT technique.^[14]^ However, these techniques also have their respective disadvantages. The early rehabilitation of the AMP technique tends to be stalled on account of inadequate socket length, risk of tunnel back wall blowout, and relatively weak graft fixation; consequently, it involves a higher probability of experiencing ACL reconstruction failure and tunnel expansion in the long term.^[15]^ The OI technique can create a longer femoral tunnel, but the steep slopes of the graft and tunnel can lead to graft damage and tunnel expansion.^[16]^ Moreover, this procedure requires an additional incision of skin, which may be esthetically unpleasing.^[17]^

Nowadays, the standard TT (sTT) technique has been developed into a new technique termed as the modified TT (mTT) technique, which is effective in creating oblique femoral tunnels with fewer incisions.^[18]^ However, because the center of the femoral tunnel is often located in the AMP bundle footprint, it is extremely challenging to place the femoral tunnel in the center of the ACL footprint in the lateral femoral condyle.^[19]^

Two systematic reviews have investigated the clinical outcomes of the TT technique and ID technique,^[13,20]^ but neither of them considered the difference in different techniques to derive more comprehensive results. Therefore, we still lack a clear picture regarding the difference in efficacy and safety among the sTT, AMP, and OI surgical techniques in ACL reconstruction. Additionally, the relative merits of the mTT technique are controversial. In view of the current context, there seems to be an urgent need to evaluate the present knowledge base regarding all the available techniques in ACL reconstruction.

Network meta-analysis is widely used for comparing multiple regimens in a single analysis simultaneously, and the results from the combination of indirect and direct evidence can be further ranked in order to identify the most advantageous surgical technique.^[21,22]^ In this paper, direct evidence refers to the evidence acquired from randomized control trials (RCTs), while indirect evidence refers to the evidence acquired by 1 or more common comparators.^[21]^ The conventional meta-analysis of RCTs is deemed the best and the most common method to acquire evidence of healthcare decisions.^[23]^ Compared with pairwise meta-analysis, network meta-analysis is able to allow for visualization of more evidence, and evaluate treatments that have never been compared head-to-head in a controlled setting.^[24]^ Therefore, we intend to conduct a network meta-analysis to comparatively evaluate the efficacy and safety among the sTT, mTT, AMP, or OI surgical techniques in ACL reconstruction.

## 2 Methods

This meta-analysis aims to compare the clinical results between the sTT, mTT, AMP, and OI surgical techniques in ACL reconstruction by following the Preferred Reporting Items for Systematic Review and Meta-Analyses Protocols (PRISMA-P).^[25]^ We have registered this study in the Open Science Framework (OSF) registries (osf.io/uzahs).

### 2.1 Eligibility criteria

#### 2.1.1 Type of study

We will include prospective RCTs focusing on the comparisons between sTT, mTT, AMP, and OI techniques for ACL reconstruction.

#### 2.1.2 Type of patients

The patients involved in the included RCTs should be those who had undergone the primary arthroscopic ACL reconstruction.

#### 2.1.3 Type of interventions

AMP, OI, sTT, or mTT of femoral tunnel drilling.

#### 2.1.4 Type of outcomes

1) International Knee Documentation Committee subjective/objective score;
2) Lachman test;
3) Lysholm score;
4) laxity of knee joint;
5) pivot-shift test;
6) Tegner activity scale, and
7) the summed number of events of infection, effusion, and graft rupture for each technique.

### 2.2 Data sources

EMBASE, PubMed, and the Cochrane Library will be searched through to retrieve the relevant literature up to March 2020. Meanwhile, the references of the published reviews and retrieved studies will also be evaluated to identify additional eligible studies.

### 2.3 Search strategy

The key search terms or medical subject heading terms will be used to perform the literature search in the 3 databases aforementioned. The PubMed search strategy is shown in Table 1. The search terms will be adapted appropriately to conform to different syntax rules of different databases.

**Table 1:**
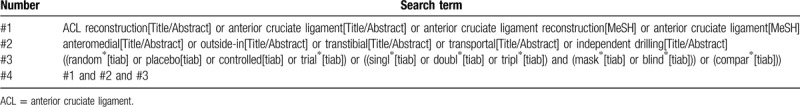
Electronic search strategy in PubMed.

### 2.4 Study selection

**Figure 1:**
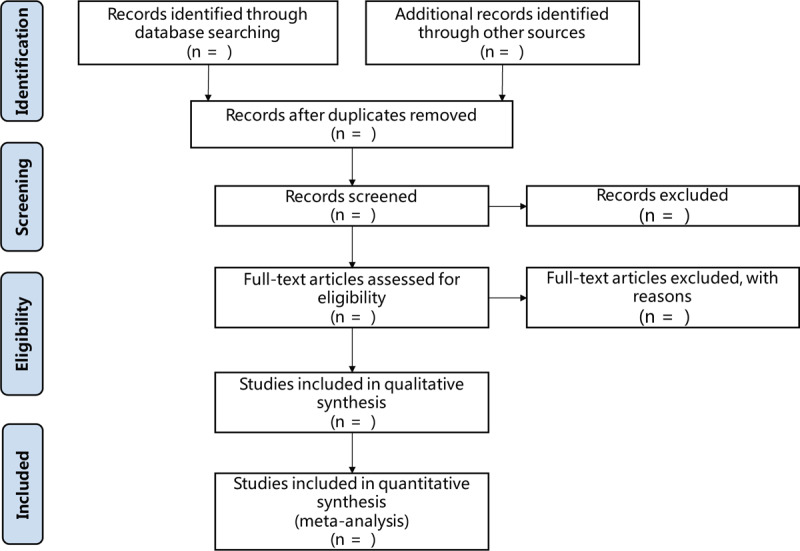
Literature screening process.

Two authors will be responsible for investigating the titles and abstracts of all retrieved records independently to identify eligible trials. Two reviewers will independently screen each record retrieved by EndNote, and review full text of all potential literatures for further assessment to exclude irrelevant studies or determine eligibility. There will be no restriction on the publication date or language. The detailed selection process will be shown in the Preferred Reporting Items for Systematic Reviews and Meta-Analyses flow diagram. (Fig. 1) Disagreements between the 2 authors, if any, will be resolved by discussing with a third author.

### 2.5 Data extraction

The 2 authors will extract the following data independently using a standardized collection form: publication information (name of author, country of origin, year of publication, and name of journal); participant information (sample size, age, sex, and follow-up duration); intervention information (surgical technique); outcome information (International Knee Documentation Committee subjective/objective score, Lachman test, Lysholm score, laxity of knee joint, pivot-shift test, Tegner activity scale, as well as the number of events of infection, effusion, and graft rupture). If any concerned information is not reported in an included study, the missing data will be acquired by alternative methods as far as possible, including contacting the author(s) directly through email.

### 2.6 Risk of bias of individual studies

Two reviewers will be designated to assess the quality of included RCTs independently by utilizing the Cochrane risk of bias assessment tool.^[26]^ As specified by Cochrane Handbook V.5.1.0, the following sources of bias will be considered: random sequence generation, allocation concealment, participant blinding, outcome assessor blinding, incomplete outcome data, selective reporting, and other sources of bias. Each domain will be rated as high, low or unclear risk of bias as appropriate. The 2 reviewers will resolve any disagreements through discussion, and a third reviewer may be involved if no consensus is reached.

### 2.7 Geometry of the network

A network plot will be created to present the diagram of the intervention network for the comparison across trials using STATA software (version 15.0, StataCorp, College Station, TX). In the network geometry, each node represents an intervention, while each edge describes the head-to-head comparison between 2 interventions. The size of a node reflects the sample size for the corresponding intervention; the thickness of an edge shows the number of trials that are included for comparison.

### 2.8 Statistical analysis

A Bayesian hierarchical framework will be used to evaluate the comparative efficacy among different fixation devices. The Bayesian network meta-analysis method, described in previous publications,^[27,28]^ can effectively increase the number of studies included for each comparison and narrow the width of credible interval of the estimate.^[29,30]^ The posterior density for an unknown variable will be estimated by applying the Markov Chain Monte Carlo method using WinBUGS software (version 1.4.3, MRC Biostatistics Unit, Cambridge, UK), and the heterogeneity of the included studies will be evaluated by Cochrane Q test and I^2^ statistics. If *P* value > .05 of the Q statistics and I^2^ value < 50%, fixed effects models will be used to pool the data during meta-analysis; otherwise, random effects models will be used instead. All figures that describe the results will be generated by STATA software.

#### 2.8.1 Subgroup analysis

If variability exists among the studies with regard to graft types or devices used for graft fixation, subgroup analysis will be performed to investigate whether the results were significantly different.

#### 2.8.2 Sensitivity analysis

If necessary, sensitivity analysis will be performed to evaluate data reliability on the basis of the methodological quality.

#### 2.8.3 Inconsistency examination

The consistency assumption for the whole network will be evaluated by the design-by-treatment interaction model.^[31]^ If there are >3 interventions, the node-splitting method will be used to investigate the inconsistency between direct and indirect evidence for every paired comparison.^[32,33]^

#### 2.8.4 Ranking of effects

The surface under the cumulative ranking curve will be calculated and used to rank all the interventions in accordance with each outcome that accounts for the uncertainty in the treatment effect.^[34]^

#### 2.8.5 Assessment of publication bias

Begg and Egger funnel plot will be performed to evaluate the publication among included trials in accordance with the publication bias that may reduce the evidence intensity.^[35,36]^ The existence of publication bias will be confirmed if an asymmetric pattern is observed, and the bias will be evaluated and explained in detail through discussion.

### 2.9 Quality of evidence

Evidence quality will be evaluated using the Graduates Assessments, Development and Evaluation approach, which assesses the quality of evidence for each outcome by 5 considerations (study limitation, consistency of effect, imprecision, indirectness, and publication bias). Specifically, the evidence quality will be classified into 4 levels (high, medium, low, and very low).^[37]^

### 2.10 Patients and public participation

As a systematic review and network meta-analysis, patients, and the public will not be directly involved in the design or planning of this study.

### 2.11 Ethics and communication

Since this systematic review and network meta-analysis will be conducted with only published studies and involve no private and confidential patient data, there will be no ethical review required. We aim to provide available evidence for surgeons to identify the most appropriate surgical technique for ACL reconstruction. The findings of this review will be published in a peer-reviewed journal. No ethical issues will be raised.

## 3 Discussions

ACL injury is the most commonly-seen ligament injury of the knee joint and ACL reconstruction is most often recommended for the treatment of ACL injury. Success of the ACL reconstruction needs to be supported by proper placement of femoral tunnels so as to optimize graft alignment, and the treatment outcome can be measured by clinical, functional, and patient-oriented assessments through physical examination and questionnaire survey. At present, arthroscopic ACL reconstruction techniques can be classified into the sTT, mTT, AMP, and OI techniques. Although some systematic reviews have investigated the clinical outcomes of the TT technique and ID technique, there still lacks a clear picture regarding the comparative clinical outcomes among the sTT, mTT, AMP, and OI surgical techniques. Therefore, a comprehensive evaluation of the present knowledge base regarding all these methods in ACL reconstruction is important.

The conventional meta-analysis of RCTs is usually considered as the best and the most common approach of acquiring evidence of healthcare decisions. Compared with pairwise meta-analysis, the network meta-analysis can simultaneously compare multiple regimens in a single analysis, and the results from both direct and indirect evidence can be further ranked in order to identify the most advantageous surgical technique. In view of this, we intend to conduct this systematic review and network meta-analysis to comprehensively evaluate the efficacy and safety among the sTT, mTT, AMP, or OI surgical techniques in ACL reconstruction, hoping to provide clinical practitioners and guideline makers with useful reference.

## Author contributions

**Conceptualization:** Yilun Wang and Dongxing Xie.

**Data curation:** Zhenglei Zhu, Ziying Wu, Hongyi He, Haochen Wang, Wei Li.

**Methodology:** Zhenglei Zhu, Ziying Wu, Hongyi He, Haochen Wang, Wei Li.

**Writing – original draft:** Ning Wang and Dongxing Xie.

**Writing – review & editing:** Yilun Wang and Dongxing Xie.
